# Exeporfinium chloride (XF-73) nasal gel dosed over 24 hours prior to surgery significantly reduced *Staphylococcus aureus* nasal carriage in cardiac surgery patients: Safety and efficacy results from a randomized placebo-controlled phase 2 study

**DOI:** 10.1017/ice.2023.17

**Published:** 2023-07

**Authors:** Julie E. Mangino, Michael S. Firstenberg, Rita K.C. Milewski, William Rhys-Williams, James P. Lees, Aaron Dane, William G. Love, Jesus Gonzalez Moreno

**Affiliations:** 1 Division of Infection Diseases, Department of Internal Medicine, The Ohio State University, Columbus, Ohio, United States; 2 William Novick Global Cardiac Alliance. Aurora, Colorado, United States; 3 Department of Surgery, Yale School of Medicine, New Haven, Connecticut, United States; 4 Destiny Pharma, Brighton, United Kingdom; 5 Danestat Consulting Ltd, Macclesfield, United Kingdom

## Abstract

We studied 83 cardiac-surgery patients with nasal *S. aureus* carriage who received 4 intranasal administrations of XF-73 nasal gel or placebo <24 hours before surgery. One hour before surgery, patients exhibited a *S. aureus* nasal carriage reduction of 2.5 log_10_ with XF-73 compared to 0.4 log_10_ CFU/mL for those who received placebo (95% CI, −2.7 to −1.5; *P* < .0001).

Methicillin-susceptible *Staphylococcus aureus* (MSSA) and methicillin-resistant *Staphylococcus aureus* (MRSA) are the most common causes of healthcare-associated infections in hospitals worldwide.^1^ Between 15% and 30% of healthy adults are nasally colonized with MSSA and 1%–3% are colonized with MRSA.^2^ *S. aureus* carriers are at a higher risk of staphylococcal infections after surgery than noncarriers and are up to 9 times more likely to develop surgical-site infections (SSIs).^3,4^

In a large, randomized, controlled study comparing topical, nasal mupirocin versus placebo for a variety of surgical procedures, mupirocin prophylaxis decreased the rate of *S. aureus* SSIs among *S. aureus* carriers: 3.7% in mupirocin group versus 5.9% in controls.^5^ A consensus of the Surgical Infection Society (SIS), Infectious Disease Society of America (IDSA), and the Society for Healthcare Epidemiology for America (SHEA) recommends nasal decolonization in those with documented *S. aureus* colonization for high-risk cardiac and orthopedic surgeries.^6^

Although not a formal indication, mupirocin is used as a topical antibiotic prior to surgery for nasal decolonization of MSSA and MRSA, worldwide.^7^ Implementation is affected by challenges with screening, ordering and administration, course duration, mupirocin resistance, and issues with adherence because it requires a multidisciplinary approach coupled with patient education.^4,7^

Exeporfinium chloride (XF-73) is a dicationic porphyrin derivative with rapid potent bactericidal properties and a low propensity for engendering bacterial resistance.^8,9^ It is being developed as a gel for nasal *S. aureus* decolonization to prevent postoperative staphylococcal SSIs. This phase 2 study was designed to assess microbiological efficacy and safety of XF-73 in reducing nasal *S. aureus* prior to open-chest cardiac surgery.

## Methods

In this multicenter, randomized, placebo-controlled, phase 2 study, we assessed the effect of nasal XF-73 versus placebo on *S. aureus* nasal burden in patients undergoing cardiac surgery (Clinical trial identifier: NCT03915470). The study was conducted between August 29, 2019, and March 29, 2021, under good clinical practice guidelines (ICH E6 GCP) with all regulatory and ethical approvals in place. We included male and female patients, aged 18–75 years, who were due to undergo cardiac surgery. Patients were initially screened using a PCR assay (Cepheid Xpert *S. aureus* Nasal Complete Assay, Cepheid, Sunnyvale, CA) to identify nasal *S. aureus* carriage to be eligible to participate.

Patients were randomized (1:1) to receive 0.2% (w/w) XF-73 or color-matched placebo as 3 doses (ie, the primary end point), 1 dose immediately before cardiac surgery, and a fifth dose after surgery (ie, 5 doses in total). Of the 5 doses, 4 were administered <24 hours prior to surgery. Destiny staff trained research staff in nasal swab collection and dose administration; nasal swab cultures were obtained just prior to the next application of XF-73 or placebo by research staff. All nasal swabs were analyzed in a central laboratory within each country and the same techniques were followed in all laboratories. Nasal swabs were plated onto BBL ChromAgar plates. S. *aureus* colonies were converted to log_10_ CFU/mL to measure nasal burden, at baseline and immediately before and after surgery. Perioperative antibiotic use and preoperative antiseptic skin decolonization were left up to each center’s standard of care and were recorded. Antiseptic skin decolonization refers to “full-body skin decolonization” implemented preoperatively (eg, at home or on the inpatient unit prior to arriving to the operating room), with at least 1 application.

The primary end point of interest in this study was change in *S. aureus* log_10_ CFU/mL from baseline to 1 hour before surgery (after 3 doses), in the micro-ITT set (ie, patients with confirmed *S. aureus* nasal carriage at baseline). Safety measures included adverse events; ear, nose, and throat (ENT) examinations; and a brief smell-identification test (B-SIT, Sensonics International, NJ). Primary analyses of change in log_10_ CFU/mL from baseline to 1 hour pre-surgery were performed using an analysis of covariance (ANCOVA) model including baseline log_10_ CFU/mL as a covariate.

## Results

Patients were recruited in the countries of Georgia (89.2%, 9 centers), Serbia (7.2%, 2 centers), and the United State (3.6%, 2 centers). Most of these patients were male (75.9%). The most common surgeries were coronary artery bypass graft (63.8%), mitral valve replacement and/or repair (16.9%), and aortic valve replacement (16.9%). A foreign implant was placed in 39.8% of patients in the micro-ITT primary efficacy set; 100% of patients received all 5 doses. MSSA colonization occurred in 96.8% of patients, and 3.2% had MRSA.

**Fig. 1.**
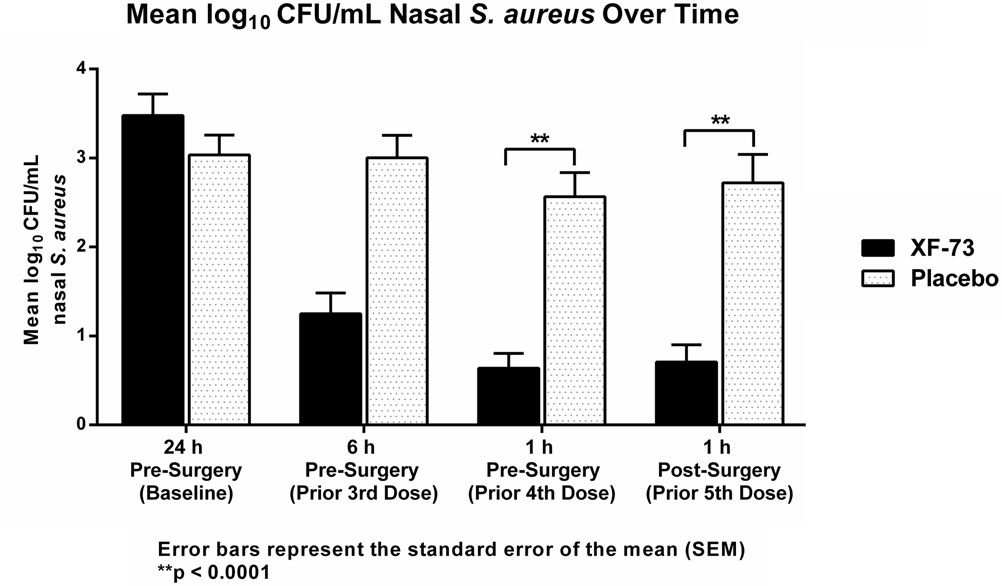
Change in burden of nasal *S. aureus* before and after surgery. Note. CFU, colony-forming units; h, hour.

The baseline nasal *S. aureus* log_10_ CFU/mL were similar in both groups (Fig. 1). After 3 applications of XF-73 nasal gel over <24 hours (1 hour prior to surgery, the primary end point), we detected a greater decrease in nasal *S. aureus* observed in the XF-73 arm (−2.842 log_10_ CFU/mL) versus placebo (−0.469 log_10_ CFU/mL). The adjusted least-squares mean difference between the 2 groups was −2.1 log_10_ CFU/mL and was statistically significant (95% CI, −2.7 to −1.5; *P* < .0001).

Within 1 hour of incision closure, the decrease in *S. aureus* was also significantly greater in the XF-73 cohort compared to the placebo group, with a least-squares mean difference of −2.2 log_10_ CFU/mL (95% CI, −2.7 to −1.6; *P* < .0001) (Fig. 1, postsurgery).

**Fig. 2.**
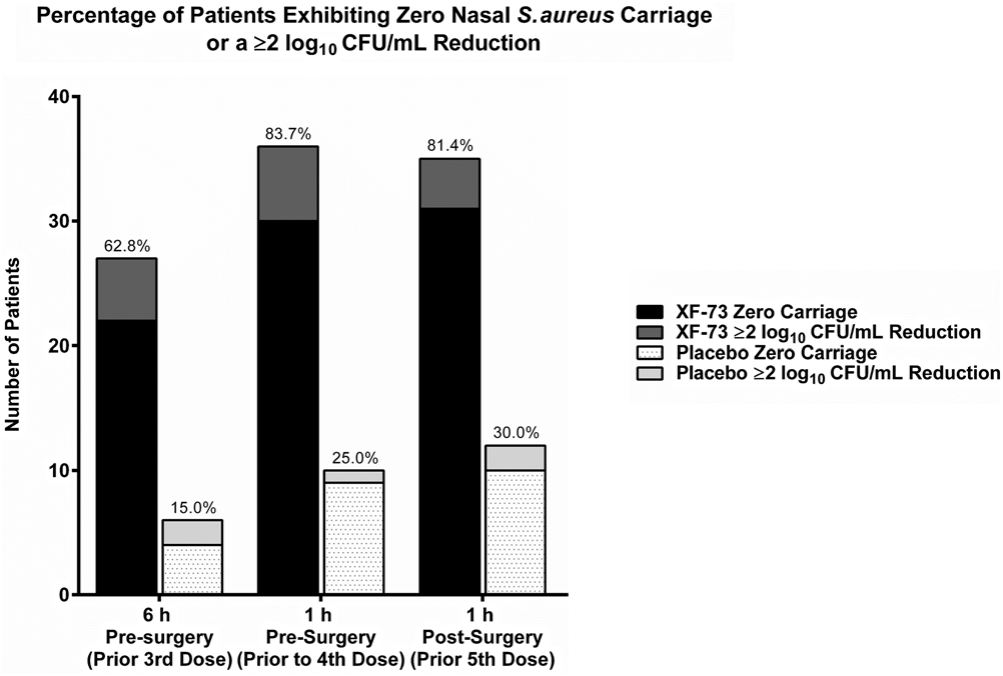
Percentage of patients with zero nasal *S. aureus* carriage or ≥2 log_10_ CFU/mL reduction. Note. CFU, colony-forming units; h, hour.

When assessing the percentage of patients exhibiting zero nasal *S. aureus* carriage, (decolonization), or a ≥2 log_10_ CFU/ml reduction, 83.7% of patients treated with XF-73 met this metric within 1 hour prior to surgery (Fig. 2). For antiseptic skin decolonization, 12 of 13 centers used chlorhexidine gluconate, benzalkonium chloride, or “other” as their standard of care. One site recorded no skin decolonization.

No SSIs were identified during the study or follow-up periods of 1 month or 3 months (for those with a foreign implant). Overall, 95.2% of patients received prophylactic skin decolonization preoperatively.

No treatment-emergent adverse events (TEAEs), laboratory or other safety findings were considered related to either arm in the 124 patients evaluated as the safety population. In the XF-73 and placebo groups, respectively, the most frequently reported TEAEs were pleural effusion (16 and 14 patients), anemia (5 and 6 patients), pericardial effusion (5 and 5 patients), and atrial fibrillation (5 and 4 patients).

No local reactions at the application site (ie, anterior nares) occurred according to an ENT specialist and there were no significant changes to sense of smell (B-SIT) during the study.

## Discussion

Administration of XF-73 nasal gel <24 hours prior to surgery rapidly and significantly reduced nasal *S. aureus* burden preoperatively. The primary end point of this phase 2 study was met: XF-73 nasal gel reduced nasal *S. aureus* burden from baseline to 1 hour prior to surgery, with a highly statistically significant reduction of 2.1 log_10_ CFU/mL more than placebo. No safety or tolerability issues were identified.

The most widespread topical antibiotic recommended within SSI prevention guidelines for nasal *S. aureus* decolonization is mupirocin. However, guidelines also carry a warning to limit widespread use of mupirocin, due to concerns of generating mupirocin-resistant strains of *S. aureus,* which could dominate.^7^ Global reports indicate that mupirocin-resistant *S. aureus* prevalence has increased to 7.6% and mupirocin-resistant MRSAs have significantly increased to 13.8%.^10^ In this study, XF-73 nasal gel had a favorable safety profile in this patient population without any TEAE’s related to the study drug nor placebo. No postoperative SSIs were observed in either study arm.

This study had several limitations. Infection prevention training was not standardized across countries; a large proportion of the recruitment population was from Georgia; and only those patients with intravascular implants were followed for 90 days.

The advantages of XF-73 nasal gel include (1) a short presurgical dosing period required, (2) rapidity of *S. aureus* decolonization, and (3) previously demonstrated remote likelihood of generating staphylococcal strains that are resistant to XF-73.^9^
